# Long-term complications of cosmetic iris implants

**DOI:** 10.1186/s12886-022-02650-1

**Published:** 2022-11-30

**Authors:** Juan Queiruga-Piñero, Tomas Parra-Rodriguez, Ignacio Rodriguez-Una

**Affiliations:** 1 Instituto Oftalmologico Fernandez-Vega, Avda. Dres. Fernandez-Vega, 34, 33012 Oviedo, Spain; 2 Fundacion de Investigacion Oftalmologica, University of Oviedo, Oviedo, Spain

**Keywords:** Corneal edema, Iris cosmetic implant, Intraocular pressure, Pigment dispersion syndrome, Iris-trabecular contact, Gonioscopy, Irido-corneal angle

## Abstract

**Background:**

Additive cosmetic implants (NewColorIris, Kahn Medical Devices, Panama City, Panama) are placed in the anterior chamber, in order to externally change iris color. There is a lack of robust clinical long-term prospective studies regarding the safety of these devices, as they have been related to the early-onset presentation of corneal decompensation, elevated intraocular pressure (IOP), uveitis and hyphema. However, in this case report some mild complications started to manifest unexpectedly late: 15 years after an uneventful procedure.

**Case presentation:**

A 41-year-old Caucasian woman presented with blurred vision in both eyes over the last 6 months. Fifteen years earlier, she had undergone bilateral implantation of additive iris implants for aesthetic purposes, without any complication or ocular trauma during the follow-up. Ocular examination showed bilateral mild corneal edema, iris atrophy, and presence of pigment in the endothelium. Increased IOP (28 mmHg) was identified in the right eye. Anterior segment optical coherence tomography (AS-OCT) confirmed the decentration of the iris implant from the pupillary axis in that eye. Gonioscopy demonstrated pigment dispersion in both eyes, as well as a tendency to bilateral angle closure, that was also illustrated by AS-OCT analysis. Endothelial cell count was 1268 cells/mm^2^ in the right eye and 1122 cells/mm^2^ in the left eye.

The presence of both implants was affecting corneal endothelium and anterior chamber angle in both eyes, and additionally, the decentration of the device in the case of the right eye led to secondary ocular hypertension in that eye.

**Conclusions:**

Cosmetic implants in contact to the iris can remain quiescent for years, leading to possible complications that can present even in the long-term. The degree of implant decentration, the stage of angle closure disease and the magnitude of pigment dispersion may be some important factors related to the onset time of complications in these cases.

## Background

Prosthetic iris implants have been used in functional, traumatic and congenital abnormalities of the iris, safely and effectively decreasing flashes and light sensitivity [1]. These iris implants should not be confounded with the one commercialized under the name of NewColorIris (Kahn Medical Devices, Panama City, Panama). While prosthetic implants replace the damaged iris, this additive cosmetic implant is placed in the anterior chamber (AC) in front of the iris, changing its natural color [2]. There are no studies regarding the safety of this type of implant in the long term and their use has not been approved by the Food and Drugs Administration (FDA) in the United States. Similarly, in Europe they lack the CE marking. In addition, several serious complications have been reported including corneal decompensation, elevated intraocular pressure (IOP), uveitis and hyphema [3].

While those complications tend to manifest in the early-time follow-up, this case presents a patient with a late onset of several complications, later than a decade after surgey.

## Case presentation

**Fig. 1:**
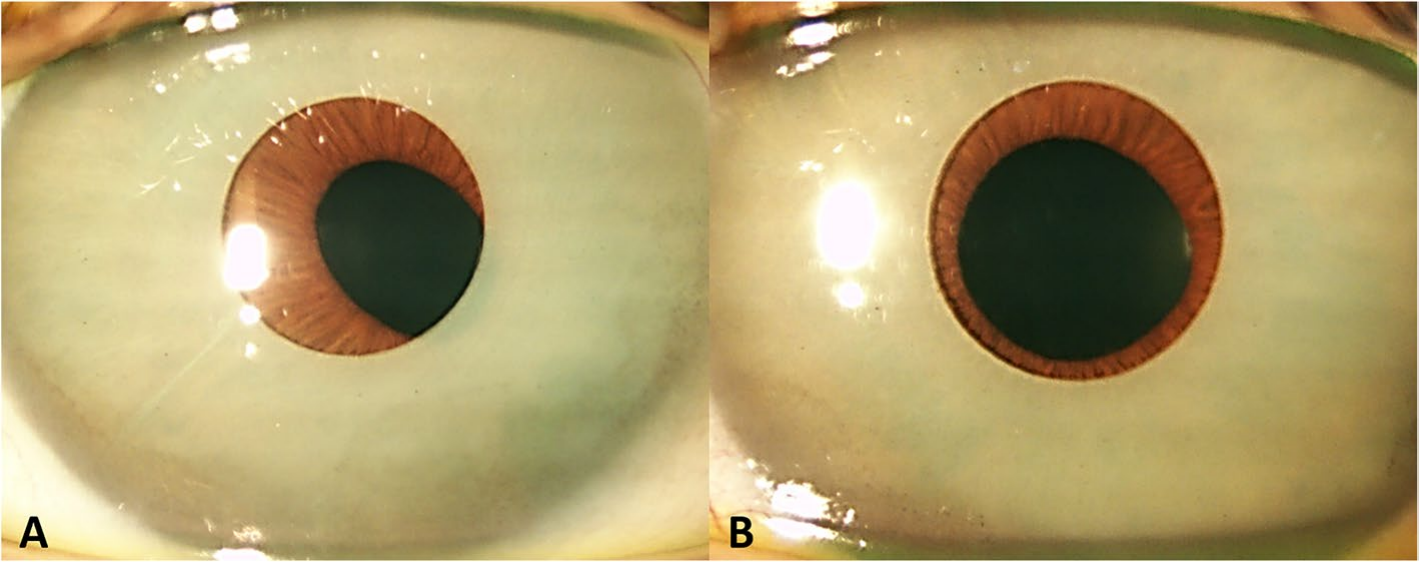
Cosmetic iris implants located in the anterior chamber with iris pigment deposited in the cornea. The right implant (**A**) was significantly displaced to the temporal and superior sectors and was not alligned with the pupillary axis; the pupil was slightly ovalized. The left implant (**B**) was more centered and the pupil was round

A 41-year-old Caucasian woman referred 6 months of bilateral blurred vision, more intense when she woke up. She had undergone surgery for implantation of cosmetic NewColorIris devices in both eyes 15 years before, without any complication or ocular trauma during that time (Fig. 1).

Ocular examination confirmed low hyperopia (+ 0.75 D) in both eyes and she did not wear contact lenses (CL). Her best-corrected visual acuity was 20/20 in both eyes. The slit lamp examination showed bilateral and symmetric slight corneal edema with early decompensation, epithelial bullae and presence of pigment in the endothelium (specular microscopy showed a low endothelial cell count in the right eye (1268 cells/mm^2^), as well as in the left one (1122 cells/mm^2^). Silicone cosmetic implants in front of the iris were also observed in the AC with a central hole of 3.4 mm that did not match the pupillary axis in the right eye (Fig. 1). Although iris evaluation was limited due to those implants, iris atrophy was observed by backlighting.

**Fig. 2:**
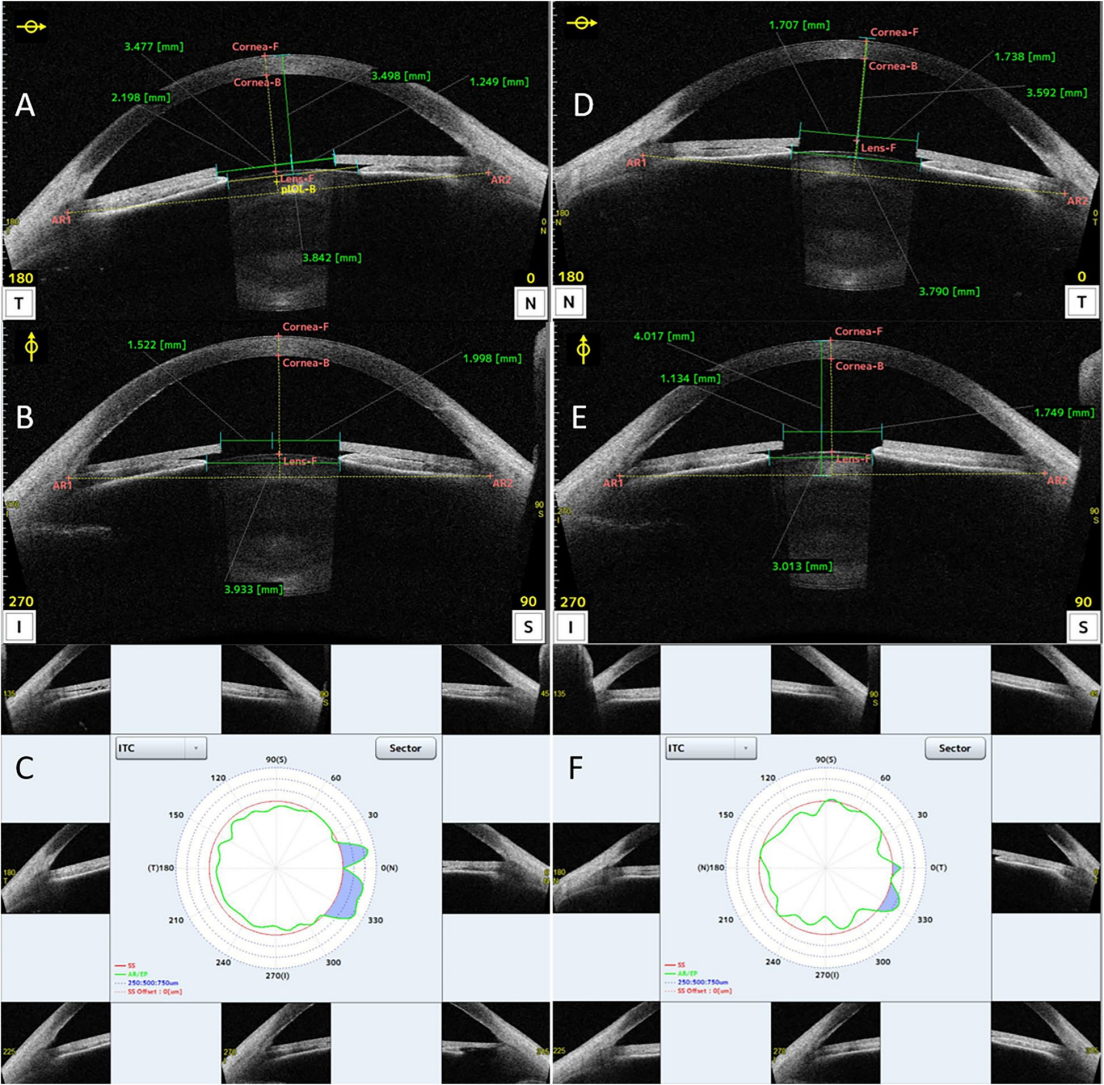
AS-OCT analysis (CASIA2, Tomey). Iris implants were placed on the anterior layer of the iris, exceeding the pupil border and contacting with the irido-corneal angle. The green vertical line represents the pupillary axis. By using the calliper system provided by CASIA2, the horizontal (0°-180° axis, **A** and **D**) and vertical (90°-270° axis, **B** and **E**) distance between this axis and the internal edges of the central hole of the iris implants could be measured. On each axis, 2 segments were measured (nasal/temporal, superior/inferior), and sustracting the longest of them from the central hole radius, the magnitude of the decentration on every direction was determined. Iris-trabecular contact (ITC) analysis in the right eye (**E**) and the left eye (**F**): blue area shows the contact of the iris with the trabecular meshwork, confirming some areas of peripheral anterior synechiae in both eyes (SS: scleral spur, red line; AR/EP: trabecular iris endpoint contact marked by an observer analyzing the image, green line)

The position of both iris implants was assessed in detail by the means of anterior segment optical coherence tomography (AS-OCT, CASIA2, Tomey Corporation, Nagoya, Japan), and decentration was measured with several scans (Fig. 2). The decentration of the iris implant from the pupillary axis in the right eye was 475 μm to the temporal sector and 238 μm superiorly (Fig. 2A, B). The iris implant in the left eye was more centered: it had moved 308 μm superiorly but only 15 μm to the temporal quadrant (Fig. 2D, E).

IOP measurements obtained with Goldmann applanation tonometry were 28 mmHg and 18 mmHg in the right eye and in the left eye, respectively. Central corneal thickness was 588 μm in the right eye and 559 μm in the left eye.

**Fig. 3:**
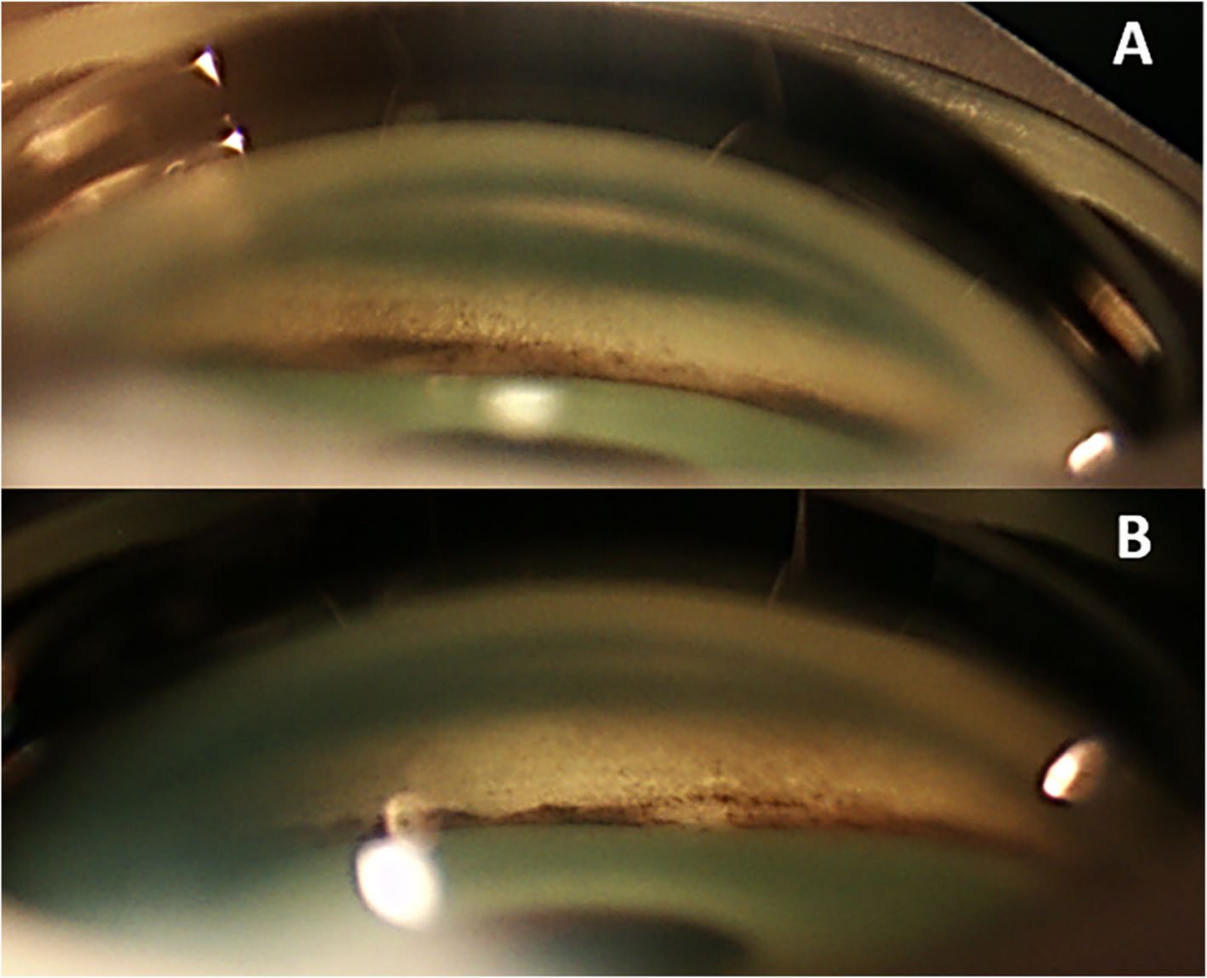
Gonioscopic image of the inferior sector of the iridocorneal angle (**A**: right eye; **B**: left eye). Marked and homogeneus pigmentation was found, suggesting pigmentary dispersion syndrome. Extense iridotrabecular contact can be observed, representing a possible sign of angle closure

Gonioscopy (performed with a Posner gonioprism) demonstrated a tendency to angle closure in both eyes (grade II in the superior, inferior and temporal quadrants and 0 in the nasal quadrant, according to Shaffer’s grading system to assess angle opening), with a large amount of pigment at the angle, more increased at VI o’clock (Fig. 3). Those findings were also identified by the means of the iris-trabecular contact (ITC) analysis provided by AS-OCT: the ITC index was 18.3% in the right eye and 13.3% in the left eye, with nasal areas of possible peripheral anterior synechiae (PAS) in both eyes (Fig. 2).

Fundal examination revealed no pathological findings and the optic discs were normal, with a cup-to-disc ratio of 0.4 in both eyes. Humphrey computerized perimetry performed with SITA standard 24–2 strategy (Carl Zeiss Meditec, Dublin, CA, USA) showed no perimetric defects in either eye. The retinal nerve fiber layer thickness measured by OCT (Triton, Topcon Corporation, Tokyo, Japan) was within the normal limits in both eyes.

Based on all these results, the most likely diagnosis was bilateral corneal decompensation, as well as ocular hypertension (OHT) in the right eye, due to secondary pigment dispersion syndrome. Beta-blockers eyedrops were prescribed for her right eye, and the surgical removal of both cosmetic implants was considered as a further management option. The patient did not attend the scheduled follow-up visits.

## Discussion and conclusions

These cosmetic iris implants are advertised as a safe and permanent solution to cosmetic CL, even though the risks are slightly higher [4]. However, there are several case series publications that report multiple complications with this type of cosmetic iris implants [5]. These complications, which can become very severe, include increased IOP, pigment dispersion syndrome, uveitis, corneal edema, low endothelial cell count, uveitis-glaucoma-hyphema syndrome, glaucomatous optic neuropathy, cystoid macular edema, and suprachoroidal haemorrhage [1]. They can manifest in an early stage (during the first postoperative weeks), or later (6 months after surgery), although most complications (77.8%) occur within the first month after the procedure [3]. In the present case, the patient showed the same described complications, although the unique feature is the timeline of presentation, that was much longer than reported: 15 years after the surgery of the theoretically uncomplicated surgery. Corneal damage may be due to a dislocation of the implant in the anterior chamber when the pupil dilates [5]. This is related to some features found in this case: a decentration of the implant was observed (mostly in the right eye), leading to a low endothelial count. In addition, irregularities of the implant surface and its sharp edges observed by scanning electron microscopy can contribute to the abrasion of the corneal endothelium and iris, increasing pigment dispersion [6]. This could justify iris atrophy and the increase of pigment present in the AC and angle of the presented patient.

The presence of this pigment in the AC was reported as the main mechanism of OHT [7]: pigment deposition in the irido-corneal angle can block the trabecular meshwork, reducing aqueous humor outflow facility. According to its designers, the haptics of the implant do not exert pressure on the trabecular meshwork, Schlemm’s canal or collector channels [8]. However, the pathogenesis of increased IOP may be due to direct contact of the edges of the implant with the angle structures, eventually resulting in glaucoma [6]. One of the parameters that shows good agreement with gonioscopy in the interpretation of the extension of angle-closure is the ITC index [9]. Moreover, it may identify more closed angles since inadvertent compression of the eyeball and excessive light during gonioscopy can lead to a false opening of the angle [9]. Therefore, the value of the ITC index, as well as its graphic representation, provided by CASIA2, confirmed the contact between the implant and the angle structures in this case (Fig. 2).

The mechanisms of high IOP in this case appeared to be both, direct damage to the trabecular meshwork by the decentered implant, as well as pigment liberation secondary to mechanical rubbing of the implant and iris. That pigment dispersion could lead to deposition and damage to the irido-corneal angle. The fact that the right implant was more decentered may explain the higher IOP in that eye. A possible explanation for the late onset of the manifestations/symptoms could be that the implants were not decentered from the early postoperative follow-up, and the right one moved through time, slowly and progressively leading to the mentioned late complications. In summary, the specific complications that manifest as well as their onset time in each eye might be related to the degree of implant decentration, the stage of angle closure disease (i.e. IOP, PAS) and the magnitude of pigment dispersion. A limitation of this case report is the lack of follow-up. As the patient never attended the scheduled appointments it was not possible to assess the response to ocular hypotensive medication, and we were not able to determine the corneal and angle situation after theoretical removal of both implants.

This case shows that, although these devices can remain quiescent for several years, their complications can finally manifest, in a subtle and barely perceptible way, leading to potentially severe or irreversible damage at different structures and functions of the eye, even in the long-term. Recent reviews, that analyzed safety of this type of cosmetic implants, highlighted the most accepted theories explaining the pathophysiology of these complications [10, 11]. Corneal complications are associated to corneal edema due to loss of endothelial cells [10]: the location of the cosmetic iris implants within the anterior chamber can be related to chronic inflammation, anterior chamber turbulence, and possible dislocation associated to pupil dilation [5]. The pathogenesis of OHT and consequent glaucoma may be attributable to three main mechanisms: direct contact of the edges of the implants (that can be decentered) with the angle structures, leading to trauma of the trabecular meshwork [6]; the development of PAS and iris neovascularization; and secondary iris pigment dispersion [1, 7, 10, 11].

As the number of patients who present complications once inserted into the eye is increasing, the ethical and medical criteria should be reviewed. Currently, the risks assumed with the insertion of these devices may outweigh the benefits.

## Data Availability

The data used during the current study are not publicly available as they were obtained from the medical records of the patient. They may be available from the corresponding author on reasonable request.

## Abbreviations

AC: Anterior chamber
AS-OCT: Anterior segment optical coherence tomography
CL: Contact lens/lenses
IOP: Intraocular pressure
ITC: Iris-trabecular contact
OHT: Ocular hypertension
PAS: Peripheral anterior synechiae
