# Correction: Long-term complications of cosmetic iris implants

**DOI:** 10.1186/s12886-022-02750-y

**Published:** 2023-01-04

**Authors:** Juan Queiruga-Piñeiro, Tomas Parra-Rodriguez, Ignacio Rodriguez-Una

**Affiliations:** 1 Instituto Oftalmologico Fernandez-Vega, Avda. Dres. Fernandez-Vega, 34, 33012 Oviedo, Spain; 2 Fundacion de Investigacion Oftalmologica, University of Oviedo, Oviedo, Spain

**Correction: BMC Ophthalmol 22, 459 (2022)**

**https://doi.org/10.1186/s12886-022-02650-1**

Following publication of the original article [1], the authors have identified an error in the author name of Juan Queiruga-Piñeiro.

The incorrect author name is: Juan Queiruga-Piñero

The correct author name is: Juan Queiruga-Piñeiro

The author group has been updated above and the original article [1] has been corrected.
